# Screening and Growth Characterization of Non-conventional Yeasts in a Hemicellulosic Hydrolysate

**DOI:** 10.3389/fbioe.2021.659472

**Published:** 2021-04-29

**Authors:** Paola Monteiro de Oliveira, Daria Aborneva, Nemailla Bonturi, Petri-Jaan Lahtvee

**Affiliations:** Institute of Technology, University of Tartu, Tartu, Estonia

**Keywords:** non-conventional yeasts, hemicellulosic hydrolysate, xylose, *Rhodotorula toruloides*, *Lipomyces starkeyi*, *Kluyveromyces marxianus*, *Scheffersomyces stipitis*

## Abstract

Lignocellulosic biomass is an attractive raw material for the sustainable production of chemicals and materials using microbial cell factories. Most of the existing bioprocesses focus on second-generation ethanol production using genetically modified *Saccharomyces cerevisiae*, however, this microorganism is naturally unable to consume xylose. Moreover, extensive metabolic engineering has to be carried out to achieve high production levels of industrially relevant building blocks. Hence, the use of non-*Saccharomyces* species, or non-conventional yeasts, bearing native metabolic routes, allows conversion of a wide range of substrates into different products, and higher tolerance to inhibitors improves the efficiency of biorefineries. In this study, nine non-conventional yeast strains were selected and screened on a diluted hemicellulosic hydrolysate from Birch. *Kluyveromyces marxianus* CBS 6556, *Scheffersomyces stipitis* CBS 5773, *Lipomyces starkeyi* DSM 70295, and *Rhodotorula toruloides* CCT 7815 were selected for further characterization, where their growth and substrate consumption patterns were analyzed under industrially relevant substrate concentrations and controlled environmental conditions in bioreactors. *K. marxianus* CBS 6556 performed poorly under higher hydrolysate concentrations, although this yeast was determined among the fastest-growing yeasts on diluted hydrolysate. *S. stipitis* CBS 5773 demonstrated a low growth and biomass production while consuming glucose, while during the xylose-phase, the specific growth and sugar co-consumption rates were among the highest of this study (0.17 h^–1^ and 0.37 g/gdw*h, respectively). *L. starkeyi* DSM 70295 and *R. toruloides* CCT 7815 were the fastest to consume the provided sugars at high hydrolysate conditions, finishing them within 54 and 30 h, respectively. *R. toruloides* CCT 7815 performed the best of all four studied strains and tested conditions, showing the highest specific growth (0.23 h^–1^), substrate co-consumption (0.73 ± 0.02 g/gdw*h), and xylose consumption (0.22 g/gdw*h) rates. Furthermore, *R. toruloides* CCT 7815 was able to produce 10.95 ± 1.37 gL^–1^ and 1.72 ± 0.04 mgL^–1^ of lipids and carotenoids, respectively, under non-optimized cultivation conditions. The study provides novel information on selecting suitable host strains for biorefinery processes, provides detailed information on substrate consumption patterns, and pinpoints to bottlenecks possible to address using metabolic engineering or adaptive evolution experiments.

## Introduction

The production of chemicals by microbial cell factories using biomass-derived compounds as a substrate has been gaining research interest to develop processes, which are more sustainable compared to the ones in petroleum refineries (Löbs et al., 2017). Lignocellulosic biomass is an attractive raw material as it does not belong to the human food chain, is the most available renewable biological resource (Coz et al., 2016), and can be obtained from agricultural and forestry residues (Mancini et al., 2016). Lignocellulosic biomass is composed of cellulose (glucose homopolymer), hemicellulose (branched heteropolymer composed of pentoses, hexoses, and acetyl groups), and lignin (complex phenolic polymeric structure) (Kumar et al., 2009; Coz et al., 2016; Kucharska et al., 2018). Cellulose and hemicellulose need to be broken down for the majority of microorganisms to be utilized and converted into fuels, chemicals, or biomaterials (Kumar et al., 2009). There are several physical, chemical, physicochemical, and biological methods for the decomposition of lignocellulosic biomass. Physical pretreatment includes fragmentation methods, microwave and gamma radiation, and pyrolysis among others. Chemical methods occur in aqueous solutions and comprise the use of acid and alkaline pretreatment, ionic liquids, organic solvent, or reactions of oxidation and ozonolysis. Physicochemical methods combine oxidation with thermal treatment, such as steam or ammonia fiber explosion (AFEX). As for the biological methods, fungi, bacteria, or enzymes are used instead for the treatment of the biomass (Kucharska et al., 2018). However, the depolymerization process usually results in an accumulation of growth-inhibitory compounds, such as 5-hydroxymethylfurfural (HMF), furfural, acetic acid, and phenolic compounds (Coz et al., 2016).

Currently, demonstration and full-commercial plants are using microbial cell factories and lignocellulosic biomass-derived substrates, mainly for the second-generation ethanol production using genetically modified *Saccharomyces cerevisiae* (Isikgor and Becer, 2015; Jansen et al., 2017). *S. cerevisiae* has a high productivity and ethanol yields from different feedstocks (Sharma et al., 2020), but is not naturally able to consume xylose, the main pentose sugar in hemicellulose. Neither can *S. cerevisiae* naturally produce high amounts of industrially important building blocks and chemicals, such as oleochemicals and terpenoids. Therefore, extensive metabolic engineering might be necessary. To avoid such efforts, other yeasts have been selected as hosts for bioprocessing, due to their natural ability to convert a wide range of substrates into different products (Löbs et al., 2017) and their tolerance to the inhibitors present in the hydrolysates. The non-*Saccharomyces* strains that possess alternative metabolic routes for substrate utilization and product formation are called non-conventional yeasts (Krêgiel et al., 2017).

*Rhodotorula*, *Yarrowia*, and *Lipomyces* are genera of oleaginous yeasts, which can accumulate lipids over 20% of their cellular dry weight (Ageitos et al., 2011). Lipids and fatty acids are good substitutes for petroleum-derived oleochemicals and biofuels (Adrio, 2017). Besides lipids, yeasts belonging to the *Rhodotorula* genus can co-produce carotenoids, which are important raw materials for the food and pharma companies where they are used as colorants, antioxidants, and precursors for vitamin A (Park et al., 2018; Osorio-González et al., 2019). Lipids and carotenoid production by those microorganisms are triggered by different stresses, such as a high carbon to nitrogen ratio (C/N ratio) (Papanikolaou and Aggelis, 2011; Mata-Gómez et al., 2014). *Scheffersomyces stipitis* has been demonstrated to consume xylose and produce high levels of ethanol (Löbs et al., 2017) and xylitol (Rodrigues et al., 2011; Dasgupta et al., 2019). *Candida parapsilosis* is considered a potential producer of arabitol. Like xylitol, arabitol is a sugar alcohol that is used as a low-calorie sweetener by the food industry (Kordowska-Wiater et al., 2018). *Kluyveromyces marxianus* is one of the fastest-growing yeasts, it is thermotolerant, and is capable of producing biotechnologically relevant compounds, such as enzymes (inulinase, β-galactosidase) (Fonseca et al., 2008), ethanol (Ferreira et al., 2015), and aroma compounds, like 2-phenyl ethanol (Morrissey et al., 2015). *Cyberlindnera jadinii* is an attractive source of biomass enriched in protein and vitamins for animal feed and human consumption (Sousa-Silva et al., 2021).

Accessing the potential of nonconventional yeasts in hemicellulosic hydrolysates is an important step for the development of biorefinery models and a sustainable economy (Yamakawa et al., 2020). Therefore, in this study, nine non-conventional yeast strains (*Rhodotorula toruloides* CCT 0783, *Rhodotorula toruloides* CCT 7815, *Kluyveromyces marxianus* CBS 6556, *Scheffersomyces stipitis* CBS 5773, *Rhodotorula kratochvilovae* CBS 321, *Yarrowia lipolytica* DSM 8218, *Candida parapsilosis* DSM 70125, *Lipomyces starkeyi* DSM 70295, and *Cyberlindnera jadinii* DSM 70163) were selected and screened on a hemicellulosic C5-enriched hydrolysate from Birch (*Betula pendula*). The selected strains were further characterized, focusing on the growth and consumption patterns of hemicellulosic sugars under industrially relevant substrate concentrations.

## Materials and Methods

### Microorganisms

*Rhodotorula toruloides* CCT 0783 and *Rhodotorula toruloides* CCT 7815 were obtained from “Coleção de Culturas Tropicais” (Fundação André Tosello, Campinas, Brazil). *Kluyveromyces marxianus* CBS 6556, *Scheffersomyces stipitis* CBS 5773, and *Rhodotorula kratochvilovae* CBS 321 were obtained from Westerdijk Fungal Biodiversity Institute (Utrecht, Netherlands). *Yarrowia lipolytica* DSM 8218, *Candida parapsilosis* DSM 70125, *Lipomyces starkeyi* DSM 70295, and *Cyberlindnera jadinii* DSM 70163 were obtained from Leibniz Institute DSMZ-German Collection of Microorganisms and Cell Cultures (Braunschweig, Germany). All the strains were cultivated according to the collection instructions and stored at −80°C in 10% (v/v) glycerol.

### Lignocellulosic Hydrolysate

The C5-sugars enriched stream of Birch (*Betula pendula*) lignocellulosic hydrolysate, here named as C5-Birch, was produced via dilute acid hydrolysis and provided by Graanul Biotech OÜ (Tallinn, Estonia). The total amounts of hexoses (C6) were: 87.1 ± 9.7 gL^–1^ of glucose, 29.8 ± 2.8 gL^–1^ of galactose, and 21.0 ± 0.1 gL^–1^ of mannose. While the total amount of pentoses (C5) were: 298.1 ± 3.9 gL^–1^ of xylose and 14.1 ± 1.6 gL^–1^ of arabinose. Acetic acid and total phenols concentrations were 20.5 ± 0.5 gL^–1^ and 33.0 ± 4.1 gL^–1^, respectively. The nitrogen content of the hydrolysate was measured by the provider using ion chromatography and it was considered negligible (ammonium, 0.09 gL^−1^; NO_2_/NO_3_ was not detected). Furfural and 5-HMF concentrations were below 0.10 gL^–1^.

The pH of the hydrolysate was adjusted to 6.0 with NaOH and filtered using a Thermo Scientific^TM^ Nalgene Filter (Waltham, United States) with polyethersulfone membrane and pore size of 0.2 μm for sterilization. The hydrolysate dilutions were made using minimal medium (monopotassium phosphate, 3 gL^–1^; magnesium sulfate, 0.5 gL^–1^) and the C/N ratio was adjusted by modifying the concentration of added ammonium sulfate. No minerals or vitamins were added to the hydrolysate.

### Initial Screening of Microorganisms in C5-Birch Hydrolysate Using 96 Well Microplate Cultivation

A pre-inoculum of all nine yeast strain cultures was prepared overnight by incubation at 30°C and 200 rpm in YPD medium (yeast extract, 10 gL^–1^; peptone, 20 gL^–1^; dextrose, 20 gL^–1^) followed by cultivation in mineral medium (ammonium sulfate, 5 gL^–1^; monopotassium phosphate, 3 gL^–1^; magnesium sulfate, 0.5 gL^–1^) containing 20 gL^–1^ of glucose and supplemented with 1 mL per liter of vitamins and trace elements solutions according to Lahtvee et al. (2017). After 6 h of incubation at 30°C, the cells were washed and concentrated in a 0.9% NaCl solution. The cells were inoculated with an initial absorbance at 600 nm (OD 600 nm) of 0.1 in 150 microliters of C5-Birch lignocellulosic hydrolysate diluted with mineral medium to a total of 10 gL^–1^ of xylose plus glucose (C/N ratio of 5 mol/mol, representing a nitrogen excess condition). The cultivation was carried out using constant mixing at 30°C in the Biotek Microplate Reader Synergy| Mx (Biotek, Winooski, United States). The growth was estimated by online monitoring of their OD at 600 nm every 30 min. Experiments were carried out in at least five replicates.

### Determination of Suitable C5-Birch Hydrolysate Concentration and C/N Ratio for the Selected Yeasts

Selected strains were cultivated in shake flask experiments in diluted C5-Birch hydrolysate to determine the optimal hydrolysate concentration for further experiments. The C5-Birch hydrolysate was diluted with mineral medium (containing 5 gL^–1^ of ammonium sulfate) to a total sugar (glucose, xylose, mannose, arabinose, and galactose) concentration of 15, 28, 43, 58, 83, 99, and 145 gL^–1^ and resulting in different C/N ratios of 7, 12, 22, 30, 45, 60, and 100 (considering N-content from ammonium sulfate only), respectively. The inoculum was prepared as described in the initial screening experiments. The cultivation was carried out in 125 mL shake flasks containing a working volume of 25 mL, while yeasts were incubated under an aerobic environment at 30°C and 200 rpm. OD at 600 nm was measured using a spectrophotometer U-1800 (Hitachi, Tokyo, Japan).

### Yeast Growth and Substrate Consumption Profiling in C5-Birch Hydrolysate

For the detailed characterization of microorganisms, controlled bioreactors were used with the hydrolysate diluted to 80 gL^–1^ of total sugars (considering glucose, xylose, arabinose, mannose, and galactose) for the cultivation of *R. toruloides* CCT 7815 and 50 gL^–1^ for the *S. stipitis* CBS 5773, *L. starkeyi* DSM 70295, and *K. marxianus* CBS 6556. Pre-inoculum was prepared as described earlier using YPD medium and incubated overnight at 30°C and 200 rpm, followed by an incubation in C5-Birch hydrolysate diluted to 20 gL^–1^ of total sugars overnight. The cells were washed and concentrated in a 0.9% NaCl solution. The initial OD600 nm of the experiments was 0.5. Experiments were carried out in at least three replicates using 1 L MiniBio 1000 bioreactors (Applikon Biotechnology, Delft, The Netherlands) with an initial working volume of 800 mL. The pH was maintained at 6.0 by the addition of 1 M HCl or 2 M KOH, the temperature at 30°C, and the partial pressure of dissolved oxygen (pO_2_) was kept above 25% by varying the agitation between 400 and 800 rpm. Microorganism growth was monitored using an online biomass probe (absorbance at 1,300 nm) BugLab BE3000 Biomass Monitor (Bug Lab, Concord, CA, United States). CO_2_ production and O_2_ consumption were monitored by off-gas sensors (BlueInOne, BlueSens, Herten, Germany), and the BioXpert V2 software v. 2.95 (Applikon Biotechnology, Delft, the Netherlands) was used for data acquisition. Samples were taken regularly for dry biomass measurement, OD 600 nm, substrates, and metabolites quantification. For oleaginous yeasts, *L. starkeyi* DSM 70295 and *R. toruloides* CCT 7815, 20 mL of broth were withdrawn for lipid extraction and quantification. For *R. toruloides* CCT 7815, cultivation samples for carotenoid extraction, quantification, and identification were also collected.

### Analytical Methods

Dry cell biomass was measured gravimetrically after filtering the cultivation broth using a pore size of 0.45 μm membrane (Merck Millipore, Darmstadt, Germany) and drying at 65°C overnight. Hydrolysate composition, substrates, and metabolites concentrations were determined after centrifugation (18.000 *g* for 5 min) using HPLC (Prominence-i LC-2030C Plus, Shimadzu, Japan) equipped with a Refractive Index Detector RID-20A (Shimadzu, Japan) at 45°C. Organic acids, ethanol, and glycerol were measured using a Rezex ROA Organic Acid column (Phenomenex, Torrance, United States) at 45°C and 5 mM sulfuric acid (>99.5%) as a mobile phase. Sugars, xylitol, and arabitol were quantified using a Rezex RPM Monosaccharide column (Phenomenex, Torrance, United States) at 85°C and LC-grade H_2_O was used as a mobile phase at a flow rate of 0.6 mL/min. The concentration of phenolic compounds was estimated using a colorimetric method described in Hodge et al. (2009) using different concentrations of phenol for the construction of the calibration curve (0.025–1.0 gL^–1^). Lipids were extracted by an adaptation of the methodology of Folch et al. (1957) as described in Bonturi et al. (2015). Total lipids were determined gravimetrically. Carotenoids were extracted by the methodology described by Pinheiro et al. (2020). Concentrations of β-carotene, torulene, and torularhodin were determined using column 00F-4462-E0 Kinetex 2.6 μm C18 100 Å (Phenomenex, Torrance, United States) at 40°C. Gradient elution of 70–100% acetone within 15 min was used for the separation of carotenoids. The detector used was LC-2030/2040 PDA (Shimadzu, Kyoto, Japan) with 40°C of cell temperature. Different concentrations of β-carotene (0.5–25 mgL^–1^) were used for constructing the calibration curve. The quantification of 2-PE was done using the same HPLC equipment and column according to de Lima et al. (2020).

## Results

### Screening of Microorganisms in the Hemicellulosic Hydrolysate

**FIGURE 1:**
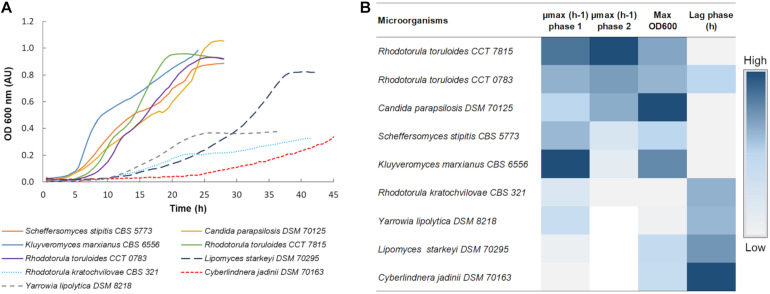
**(A)** Growth profile of all 9 yeast strains in C5-Birch hydrolysate. The OD was acquired every 30 min and the curves represent the average of at least 3 replicates, Supplementary Table 1 comprises all obtained profiles. **(B)** Heatmap of maximum achieved OD, length of lag phase, and specific growth rate for the different strains cultivated in C5-Birch hydrolysate with 10 gL^–1^ xylose plus glucose. White is used when no data is available. Data is ranked from the highest to lowest μ_max_ in the second growth phase, representing growth on xylose. Numeric data for generating the heatmap can be found in Supplementary Table 2.

Nine yeast strains were selected based on literature considering: the capacity to consume xylose; ability to grow in the presence of hydrolysate inhibitors; production of products that can be integrated into a biorefinery; and availability of omics data and synthetic biology and metabolic engineering tools. Although *Y. lipolytica* does not consume xylose naturally, this microorganism is currently the most studied non-conventional yeast with a high potential to be used as a cell factory (Ledesma-Amaro and Nicaud, 2016). Therefore, *Y. lipolytica* DSM 8218 was used in the initial screening with the other strains as a reference for their performance on glucose. Microplate cultivations of these strains were carried out using hemicellulosic hydrolysates derived from the Birch tree (C5-Birch), containing xylose as the most abundant carbon source. For the screening experiments, the hydrolysate was diluted to 10 gL^–1^ of xylose plus glucose. The strains’ performances were evaluated by monitoring their growth profile (Figure 1A and Supplementary Table 1), and identifying the length of the lag phase (hours), maximal optical density (OD600 nm), and their maximum specific growth rate (μ_max_) (Figure 1B). When a diauxic growth was observed, μ_max_ was calculated separately for both of the phases (Figure 1B and Supplementary Table 2).

Except for *L. starkeyi* DSM 70295, all the strains which were able to consume both hexose and pentose sugars presented a diauxic growth profile (Figure 1A). The first growth phase was assumed to rely on glucose consumption. In the presence of a variety of carbon sources, microorganisms tend to consume glucose first, exhibiting a carbon catabolite repression (CCR) (Gancedo, 1992). The second growth phase was considered as the xylose consumption phase, as it was the most available sugar in the used hydrolysate.

Based on the initial screening, three strains with the best performance in terms of μ_max_ were selected for the detailed characterization in C5-Birch hydrolysate; these were *K. marxianus* CBS 6556, *S. stipitis* CBS 5773, and *R. toruloides* CCT 7815. Additionally, *L. starkeyi* DSM 70295 was selected for further analysis as it did not present a typical diauxic growth profile, which might have indicated simultaneous consumption of glucose and xylose. This is a desired characteristic for a future biorefinery since it can improve the efficacy of the overall process, once a sequential consumption of the sugars can reduce the biomass yield and productivity and make processes more complex (Bothast et al., 1999; Kim et al., 2010). In our screening experiments, *K. marxianus* CBS 6556 demonstrated a short, 4-h lag-phase, had a high final OD, and the highest value of μ_max_ during the growth on glucose (0.55 ± 0.01 h^–1^). *S. stipitis* CBS 5773 had also a short lag phase (4 h) and achieved high values of μ_max_ in phase 1 and maximum OD (0.38 ± 0.02 h^–1^ and 0.89 ± 0.01, respectively). *R. toruloides* CCT 7815 had a short lag-phase and achieved the highest μ_max_ during the second growth phase, presumably on xylose (0.14 ± 0.01 h^–1^). As the *R. toruloides* CCT 7815 strain had a superior performance when compared to CCT 0783, only the former was selected for further characterization.

Although *C. parapsilosis* DSM 70125 had a short lag phase, reasonable μ_max_ in both growth phases, and high final OD, this strain was removed from further characterization. *Y. lipolytica* DSM 8218, *R. kratochvilovae* CBS 321, and *C. jadinii* DSM 70163 demonstrated relatively longer lag-phases (10.5, 11, and 20 h, respectively). *Y. lipolytica* DSM 8218 and *R. kratochvilovae* CBS 321 also did not achieve a high final OD (0.38 and 0.33, respectively) in the screening experiments, and were not further characterized.

### Concentration-Dependent Growth in the Hemicellulosic Hydrolysate

**FIGURE 2:**
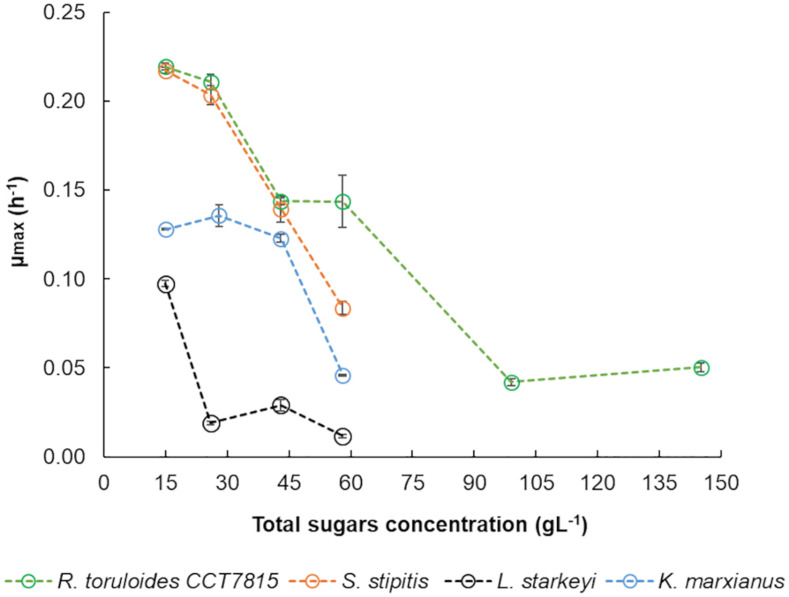
Specific growth rates calculated for *R. toruloides* CCT 7815, *K. marxianus* CBS 6556, *S. stipitis* CBS 5773, and *L. starkeyi* DSM 70295 in different dilutions of hydrolysate C5-Birch in shake flasks. Errors are expressed in standard deviation.

*Kluyveromyces marxianus* CBS 6556, *S. stipitis* CBS 5773, *L. starkeyi* DSM 70295, and *R. toruloides* CCT 7815, were cultivated in different concentrations of C5-Birch hydrolysate in shake flask experiments to analyze their total sugars and inhibitors-dependent behavior. The aim was to determine the hydrolysate concentration that could be used in bioreactor experiments. Based on growth profiles, the μ_*max*_ of each microorganism in different concentrations of C5-Birch hydrolysate were calculated (Figure 2). *K. marxianus* CBS 6556 showed a constant μ 0.13 ± 0.01 h^–1^ when cultivated in the hydrolysate diluted between 15 and 43 gL^–1^ of total sugars, however, decreasing to 0.05 ± 0.00 h^–1^ at 58 gL^–1^ of total sugars. *S. stipitis* CBS 5773 demonstrated a μ of 0.22 ± 0.01 h^–1^ in hydrolysate diluted to 15 gL^–1^ of total sugars and decreased gradually until 0.08 ± 0.01 h^–1^ when the hydrolysate dilution was increased to 58 gL^–1^ of total sugars. *L. starkeyi* DSM 70295 had the μ dropped from 0.10 ± 0.00 h^–1^ to 0.02 ± 0.00 h^–1^ when the total sugars concentration was increased above 15 gL^–1^. Regardless this significant drop in μ_max_ for *L. starkeyi* DSM 70295 above 15 gL^–1^, the hydrolysate concentration of 50 gL^–1^ was chosen for further characterization of *L. starkeyi* DSM 70295, *S. stipitis* CBS 5773, and *K. marxianus* CBS 6556.

The μ_max_ of *R. toruloides* CCT 7815 dropped from 0.21 ± 0.00 h^–1^ to 0.14 ± 0.01 h^–1^ when the total sugars concentration in the hydrolysate was higher than 43 gL^–1^. Further decrease of μ_*max*,_ at 0.05 h^–1^, was found when total sugar concentrations ranged between 99 and 145 gL^–1^. To keep a similarly expected μ_max_ between most of the yeasts, the total sugar concentration chosen for the detailed physiological characterization of *R. toruloides* CCT 7815 in bioreactors with C5-Birch hydrolysate was 81 gL^–1^. Regarding the C/N ratio, for non-oleaginous yeasts, *K. marxianus* CBS 6556 and *S. stipitis* CBS 5773, it was chosen 5 and 10 mol/mol, respectively, representing nitrogen-excess conditions. For the oleaginous yeasts, *L. starkeyi* DSM 70295 and *R. toruloides* CCT7815, the C/N molar ratio of 45 was selected to trigger lipid accumulation under nitrogen-limited conditions.

### Detailed Physiology Characterization of *R. toruloides* CCT 7815, *K. marxianus* CBS 6556, *S. stipitis* CBS 5773, and *L. starkeyi* DSM 70295 Cultivated on C5-Birch as a Substrate

The selected yeast strains were cultivated in C5-Birch hydrolysate at the chosen concentration and C/N ratio of the medium for detailed physiological characterization, under a constant pH of 6.0, and fully aerobic environment (dO_2_ > 30%). Although the ethanol and xylitol production in *K. marxianus* and *S. stipitis* are often carried out in anaerobic or microaerobic conditions, the cultivations were done aerobically as the presence of oxygen influences xylose consumption rate, demonstrating higher values under aerobic conditions (Skoog and Hahn-Hagerdal, 1990; Silva et al., 2011; Signori et al., 2014). The biomass formation, CO_2_ production, and O_2_ consumption were monitored online over the whole cultivation period. Uptake of the sugars present in the hydrolysate—xylose, glucose, galactose, mannose, and arabinose—were monitored. Acetic acid was also present in the hydrolysate and used as a substrate by all the strains characterized. All the previously mentioned parameters were used to describe the potential of the selected strains for a bioprocess development on hemicellulosic hydrolysates. Based on the substrate consumption and supporting online parameters, the growth of microorganisms was divided into different growth phases.

**FIGURE 3:**
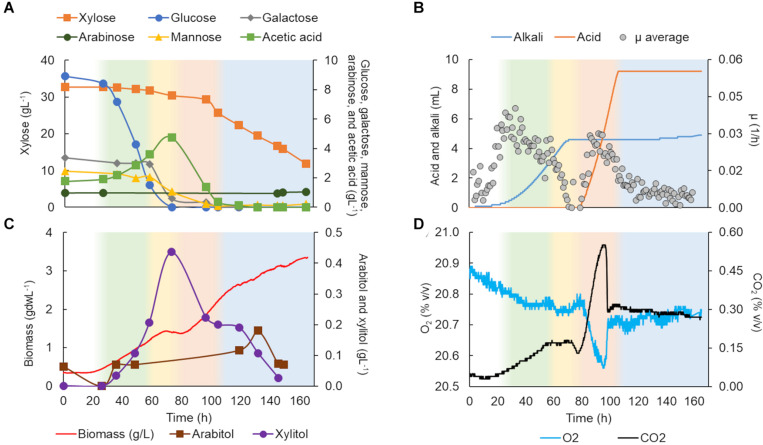
*Kluyveromyces marxianus* CBS 6556 characterization in a bioreactor in C5-Birch hydrolysate diluted to 50 gL^–1^ of total sugars. **(A)** Xylose, glucose, galactose, mannose, arabinose, and acetic acid consumption profiles (gL^–1^); **(B)** added alkali and acid profiles to maintain the constant pH and average specific growth rate (μ, h^–1^); **(C)** xylitol and arabitol production and consumption profiles (gL^−1^) and growth profile in biomass (gdwL^−1^); and **(D)** CO_2_ production and O_2_ consumption profiles. The lag phase is depicted in white; the first, second, third, and fourth growth phases are shown in green, yellow, light orange, and light blue, respectively. Supplementary Table 3 contains all data and errors expressed in standard deviation.

The growth of *K. marxianus* CBS 6556 in C5-Birch hydrolysate (total sugars 50 gL^–1^) was described in four growth phases. In the first phase (Figure 3A), glucose was consumed as the only carbon source (0.13 g/gdw*h, Table 1). Although *K. marxianus* CBS 6556 demonstrated the highest μ_max_ among the studied strains on glucose in the screening experiments, the average specific growth rate (μ) in bioreactors achieved its maximum of 0.03 h^–1^ at the beginning of the first growth phase (Figure 3B). The off-gases profile showed CO_2_ production with simultaneous O_2_ consumption (Figure 3D). Production of acetic acid also indicated sugar consumption and was consistent with alkali addition. During the second growth phase, galactose accompanied by mannose and remaining glucose were fully consumed (Figure 3A). The off-gases profiles showed constant composition regarding CO_2_ production and O_2_ consumption, but the μ dropped significantly during this phase (Figures 3B,D). The third growth phase was characterized by acetic acid consumption with a gradual increase in xylose utilization, while, surprisingly, almost no biomass was formed in this phase. The HCl inflow for the pH control (Figure 3B) was in agreement with acetic acid consumption in hydrolysates which leads to an increase in pH. This phase had one of the highest rate of substrate utilization (0.19 g/gdw*h, Table 1). A rapid increase in O_2_ consumption, CO_2_ production (Figure 3D), and average μ (0.02 h^–1^) was observed (Figure 3B). Low amounts (<0.4 gL^–1^) of xylitol and arabitol were formed and consumed during this phase, likely as a byproduct of the xylose metabolism. In the fourth phase of cultivation, the biomass curve changed its slope, and xylose was consumed simultaneously with its byproducts at a specific consumption rate of 0.07 g/gdw*h, the lowest specific rate among all phases. Throughout the experiment, no ethanol production was observed, while limited 2-phenyl ethanol was detected (up to 0.032 gL^–1^). Despite no observable stationary phase, *K. marxianus* CBS 6556 cultivation in the bioreactor was terminated at 165 h as the growth had largely stopped (Figure 3C).

**TABLE 1:** Yields and specific rates calculated from the cultivation of *R. toruloides* CCT 7815, *S. stipitis* CBS 5773, *L. starkeyi* DSM 70295, and *K. marxianus* CBS 6556 with hemicellulosic hydrolysate C5-Birch.

**Species**	**Phases**	**Substrate consumed**	**Biomass (gdwL^–^^1^)**		**Yxs (gdw/gs)**		**rs (g/gdw^∗^h)**		**μ (h^–^^1^)**	
			**Average**	**SD**	**Average**	**SD**	**Average**	**SD**	**Average**	**SD**
*R. toruloides* CCT 7815	P1	Glucose, xylose, galactose, arabinose, mannose, and acetic acid	9.50	0.62	0.26	0.02	−0.73	0.02	0.23	0.02
	P2	Xylose	23.65	1.60	0.31	0.05	−0.22	0.02	0.07	0.01
	P3	Xylose, arabitol, and xylitol	27.35	2.58	0.40	0.02	−0.01	0.00	0.01	0.01
*S. stipitis* CBS 5773	P1	Glucose	2.09	0.47	0.20	0.02	−0.10	0.00	0.02	0.00
	P2	Glucose, xylose, galactose, arabinose, mannose, acetic acid, arabitol, and xylitol	20.95	4.95	0.47	0.01	−0.37	0.03	0.17	0.01
*L. starkeyi* DSM 70295	P1	Glucose, xylose, galactose, arabinose, mannose, and acetic acid	9.05	1.16	0.51	0.06	−0.34	0.04	0.18	0.01
	P2	Xylose and arabitol	15.57	0.39	0.52	0.1	−0.25	0.03	0.13	0.03
	P3	Xylose	25.24	2.21	0.52	0.04	−0.15	0.01	0.09	0.02
*K. marxianus* CBS 6556	P1	Glucose	1.74**	0.23	0.24	0.03	−0.13	0.02	0.03**	0.00
	P2	Mannose, galactose, and glucose	2.10**	0.25	0.06	0.00	−0.21	0.01	0.01**	0.00
	P3	Xylose, acetic acid, and mannose, xylitol	3.50**	0.20	0.10	0.01	−0.19	0.01	0.02**	0.00
	P4	Xylose, arabitol, xylitol	5.08**	0.52	0.16	0.07	−0.07	0.02	0.01**	0.00

**FIGURE 4:**
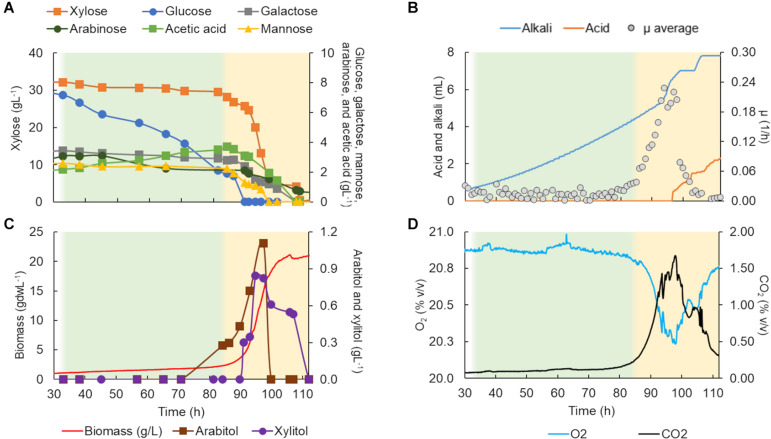
*Scheffersomyces stipitis* CBS 5773 characterization in a bioreactor in C5-Birch hydrolysate diluted to 50 gL^–1^ of total sugars. **(A)** Xylose, glucose, galactose, mannose, arabinose, and acetic acid consumption profiles (gL^–1^); **(B)** added alkali and acid profiles to maintain the constant pH and average specific growth rate (μ, h^–1^); **(C)** xylitol and arabitol production and consumption profiles (gL^–1^) and growth profile in biomass (gdwL^–1^); and **(D)** CO_2_ production and O_2_ consumed gases profiles. The lag phase is depicted in white (the graph stated at 30 h); the first and second growth phases are shown in green and yellow, respectively. Supplementary Table 4 contains all data and errors expressed in standard deviation.

**FIGURE 5:**
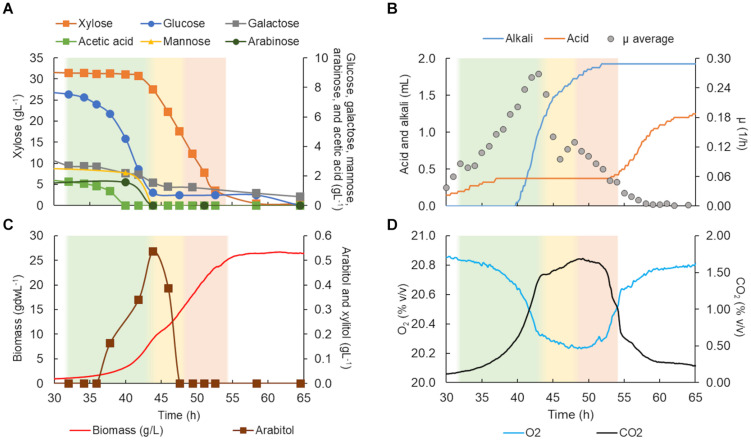
*Lipomyces starkeyi* DSM 70295 characterization in a bioreactor in C5-Birch hydrolysate diluted to 50 gL^–1^ of total sugars. **(A)** Xylose, glucose, galactose, mannose, arabinose, and acetic acid consumption profiles (gL^–1^); **(B)** added alkali and acid profiles to maintain the constant pH and average specific growth rate (μ, h^−1^); **(C)** xylitol and arabitol production and consumption profiles (gL^−1^) and growth profile in biomass (gdwL^−1^); and **(D)** CO_2_ production and O_2_ consumed gases profiles. The lag and stationary phases are depicted in white (the graph stated at 30 h); the first, second, and third growth phases are shown in green, yellow, and light orange, respectively. Supplementary Table 5 contains all data and errors expressed in standard deviation.

**FIGURE 6:**
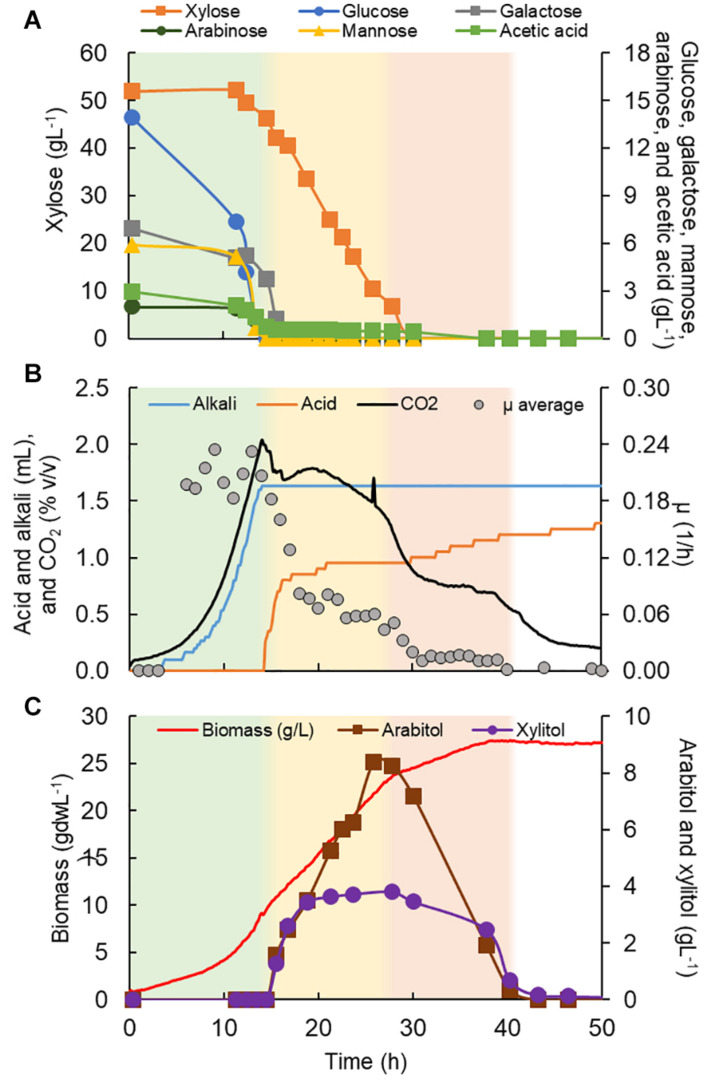
*Rhodotorula toruloides* CCT 7815 characterization in a bioreactor in C5-Birch hydrolysate diluted to 81 gL^–1^ of total sugars. **(A)** Xylose, glucose, galactose, mannose, arabinose, and acetic acid consumption profiles (gL^–1^); **(B)** added alkali and acid profiles to maintain the constant pH, CO_2_ production, and average specific growth rate (μ, h^–1^) profiles; and **(C)** xylitol and arabitol production and consumption profiles (gL^–1^) and growth profile in biomass (gdwL^–1^). The stationary phase is depicted in white; the first, second, and third growth phases are shown in green, yellow, and light orange, respectively. Supplementary Table 6 contains all data and errors expressed in standard deviation.

Different phases are shown in Figures 3–6. Errors (SD) are expressed in standard deviation (for triplicates) unless stated otherwise. **data calculated using average deviation based on duplicate measurements. Yxs – biomass yield on substrate(s); rs – specific substrate(s)uptake rate; μ – specific growth rate.

The total cultivation time for *S. stipitis* CBS 5773 in C5-Birch hydrolysate diluted to 50 gL^–1^ of total sugars and C/N ratio of 10 (mol/mol) was 112 h (Figure 4). Based on the stable concentration of the compounds present in the hydrolysate, the lag phase can be observed for up to 35 h. Two main growth phases were identified. The first phase went on for approximately 50 h and can be characterized by the lowest glucose uptake rate among all 4 strains tested in bioreactors (0.1 g/gdw*h, Table 1) which was accompanied by acetic acid production (0.56 gL^–1^). The low production of arabitol (below 0.4 gL^–1^) indicates low arabinose consumption. Off-gases, biomass, and μ profiles were following the low substrate consumption rate trend—only discrete changes were observed, such as an increase in CO_2_ production, O_2_ consumption as well as biomass formation, and instant growth rate.

During the second growth phase, all other substrates, including byproducts of xylose metabolism, were consumed simultaneously after glucose concentration had dropped below 2 gL^–1^ (Figure 4A). The average growth rate increased from 0.02 to 0.17 h^–1^ (Table 1). As expected, there was an increase in biomass and CO_2_ production, and O_2_ consumption (Figure 4D). In the second phase, all substrates were consumed simultaneously, with an average consumption rate of 0.37 g/gdw*h (Table 1). Concerning detected products, arabitol and xylitol were produced in this phase reaching maximum values of 1.1 and 0.8 gL^–1^, respectively (Figure 4C and Table 1), and were finally consumed simultaneously with xylose.

Additional experiments were carried out in shake flasks to analyze if the long lag phase demonstrated by *S. stipitis* CBS 5773 in the bioreactor was due to catabolite repression by the presence of glucose in the hydrolysate. The strain was cultivated in shake flasks using a mineral medium supplemented with 50 gL^–1^ of glucose and xylose in a ratio of 1:3.5—the same as the hydrolysate used in the bioreactor experiment. The strain started growing after 50 h of cultivation, after glucose depletion (Supplementary Figure 1). This finding suggests that the glucose catabolite repression was probably responsible for the low specific uptake rates in this phase.

*L. starkeyi* DSM 70295 grew in hydrolysate C5-Birch at 50 gL^–1^ of total sugars and a C/N ratio of 45 (mol/mol) for 55 h of cultivation when the growth ceased due to the depletion of the substrates (Figure 5). Based on the profiles of substrates consumption, average μ, and CO_2_ and O_2_ off-gases, it is possible to distinguish three different phases. After 32 h of lag phase, the first growth phase started, while the strain was co-consuming glucose, galactose, mannose, arabinose, and acetic acid with the uptake of 0.34 g/gdw*h (Figure 5A and Table 1). The growth rate increased as more substrates were co-consumed, reaching the average μ of 0.18 h^–1^ (Figures 5A,B). During this phase, it is also possible to see the start of exponential CO_2_ production and O_2_ consumption (Figure 5D). At this time, a low concentration of arabitol was produced (0.16 gL^–1^; Figure 5C), possibly by the onset of the arabinose consumption.

After 44 h of cultivation, the second phase started, which was characterized by the consumption of xylose and, additionally, arabitol—the by-product from the first growth phase—at 0.25 g/gdw*h and μ of 0.13 h^–1^ (Table 1). The CO_2_ production profile shifted once glucose and mannose were depleted, which caused a drop in μ and a shift toward arabitol consumption instead of production. The consumption of arabitol was observed simultaneously with xylose (Figure 5). In phase 3 (between 48 and 54 h of fermentation), xylose was the sole substrate and was consumed with the uptake rate of 0.15 g/gdw*h and μ of 0.09 h^–1^ (Table 1).

*R. toruloides* CCT 7815 was cultivated in C5-Birch hydrolysate diluted to 81 gL^–1^ of total sugars (60% higher concentration compared to the experiments with other strains) and a C/N ratio of 45 mol/mol was used. Based on the consumption of substrates, byproduct profiles, biomass curve, CO_2_ and O_2_ gases profile it was possible to distinguish three different growth phases with no visible lag phase (Figure 6). After 40 h of cultivation, all available carbon sources were depleted. The first growth phase was mainly characterized by the consumption of glucose, however, all the other carbon sources were consumed in the phase, when glucose levels dropped below 7.4 gL^–1^. The μ was rather constant during the first phase, showing a value of 0.23 ± 0.02 h^–1^ (Table 1). This value is higher than the one found for this strain in the previous experiment in shake flasks with the same sugar concentration (0.16 h^–1^). This can be explained by the fact that, in the bioreactors, parameters such as pH and oxygen were kept optimal throughout the cultivation. During the first phase, which lasted for 14.5 h of fermentation, all substrates except xylose were depleted (Figure 6A). The specific uptake rate of the total substrate was 0.73 ± 0.02 g/gdw*h (Table 1), the highest value obtained for substrate uptake found among the studied yeasts. μ was the highest of all phases and the biomass and CO_2_ formation indicated an exponential growth phase. No other metabolic byproduct was detected (Figure 6). The switch to xylose as a sole carbon source established the start of the second phase (between 14.5 and 28 h of fermentation). The specific xylose uptake rate was 0.22 ± 0.02 g/gdw*h (Table 1) and the xylose consumption resulted in arabitol and xylitol accumulation. The average μ dropped to 0.07 ± 0.01 h^–1^ (Figure 6B and Table 1) and remained constant until the end of the phase. A similar trend was observed for the CO_2_ formation profile. The change from the exponential profile from the first to the second phase could suggest a change in the substrate and/or reaching nitrogen-limited growth.

The depletion of xylose onset the last growth phase (third phase, 28–40 h). *R. toruloides* CCT 7815 started to consume the bioproducts xylitol and arabitol (Figure 6C) with an uptake rate of 0.01 g/gdw*h (Table 1). The specific CO_2_ production and average growth rate followed the same trend (Figure 6B). Once arabitol and xylitol were depleted the microorganism reached the stationary phase.

By the end of the cultivation, *R. toruloides* CCT 7815 had produced 1.72 ± 0.04 mgL^–1^ of total carotenoids (torularhodin: 44.4 ± 3.8%, torulene: 15.2 ± 1.2%, γ-carotene: 17.5 ± 1.0% and β-carotene: 22.9 ± 1.6%) and reached 41% of lipid content in biomass (10.95 ± 1.37 gL^–1^, C/N ratio of 45 mol/mol). Although *R. toruloides* CCT 7815 is not commonly reported amongst the typical yeasts used for xylitol production, it produced the highest titer of this compound in this work (3.80 gL^–1^). This yeast also produced the highest titer of arabitol (8.4 gL^–1^), also a low-calorie sweetener with industrial applications in medicine and agriculture (Jagtap and Rao, 2018). *L. starkeyi* DSM 70295, despite being an oleaginous yeast, accumulated only 10% of lipid content in biomass. No ethanol was detected during cultivation with *S. stipitis* CBS 5773 and this strain produced 0.85 gL^–1^ of xylitol in approximately 105 h of fermentation. Although *L. starkeyi* DSM 70295 and *S. stipitis* CBS 5773 performed poorly regarding their product titers, they achieved 25.2 and 20.9 gL^–1^ of final biomass, respectively, which might suggest their potential use as an animal feed. *K. marxianus* CBS 6556 also did not produce ethanol under the used aerobic conditions and the maximum 2-PE produced was 0.032 gL^–1^ at 132 h of cultivation.

## Discussion

From the nine initial strains used for the screening in microplate cultivations, four strains (*K. marxianus* CBS 6556, *S. stipitis* CBS 5773, *L. starkeyi* DSM 70295, and *R. toruloides* CCT 7815) were selected based on their growth performance in the lignocellulosic hydrolysate, for a more detailed characterization to better understand the growth and substrate uptake patterns of these four microorganisms in this xylose-enriched industrial hydrolysate. All these four yeast species have been reported to have the same pathway for xylose conversion. First, xylose is reduced by xylose reductase (XR) to D-xylitol using NADH or NADPH as a cofactor. XR from *S. stipitis* can use both of the cofactors (Verduyn et al., 1985), while the XR from *K. marxianus* is specific for NADPH (Zhang et al., 2013). *In silico* analysis of the XR enzyme of *R. toruloides* NP11 showed that the enzyme could use either of the cofactors (Pinheiro et al., 2020). Jagtap et al. (2019) reported a promiscuous NADPH-dependent aldose reductase in *R. toruloides* IFO 0880 capable of using xylose and galactose as substrate. An efficient NADPH regeneration is a crucial factor for the lipid accumulation and engineering of the cytosolic redox metabolism and has been demonstrated to improve lipid yields and titers (Qiao et al., 2017). Next in the pathway, D-xylitol is converted to D-xylulose by xylitol dehydrogenase (XDH). The enzyme xylulokinase (XK) converts D-xylulose to xylulose-5-phosphate which is a metabolic intermediate of the pentose phosphate pathway (PPP). There are a few reports of alternative pathways when XDH or XK are deficient or knocked out (Seiboth et al., 2003; Jin et al., 2005). XK has either been not detected or has been present in low abundance in proteomics and transcriptomics analysis of xylose-grown *R. toruloides* CCT 7815 and IFO 0880 (Pinheiro et al., 2020; Kim et al., 2021). An alternative pathway to PPP would be converting D-xylulose to D-ribulose-5-phosphate instead of D-xylulose-5-phosphate, and was recently proposed (Kim et al., 2021). Although ethanol production from hydrolysates is an important product for biorefineries, the cultivations in our study were carried out in full aerobiosis as there is a positive correlation between the presence of oxygen and higher xylose consumption rates. Anaerobiosis or microarebiosis conditions have been demonstrated to be preferred for the ethanol and xylitol production, both, in *K. marxianus* and *S. stipitis* (Skoog and Hahn-Hagerdal, 1990; Silva et al., 2011; Signori et al., 2014).

*R. toruloides* CCT 7815 demonstrated the highest μ in the tested C5-Birch hydrolysate (0.23 ± 0.02 h^–1^), consuming all the provided carbon sources the first, even at the higher total sugar concentration in the hydrolysate (81 gL^–1^). This strain also demonstrated the highest co-consumption rate (0.73 ± 0.02 g/gdw*h) during the first growth phase and the highest specific xylose consumption rate (0.22 ± 0.02 g/gdw*h). The specific xylose uptake rate measured in the current study was slightly lower than the highest rate (nitrogen-excess phase, 0.26 g/gdw*h) reported by Pinheiro et al. (2020) where the same strain was cultivated in a minimal mineral medium using 70 gL^–1^ of xylose and C/N molar ratio of 80. Although *R. toruloides* CCT 7815 had the highest specific xylose consumption rate, the μ of this strain was very similar to the one found for *L. starkeyi* DSM 70295 grown on xylose (Table 1). Unfortunately, it was not possible to separate a sole xylose phase for *S. stipitis* CBS 5773, but the specific co-consumption and μ in phase 2 were among the highest of this study (0.37 ± 0.03 g/gdw*h and 0.17 ± 0.01 h^–1^, respectively). *L. starkeyi* DSM 70295 had a similar specific co-consumption and growth rate as *S. stipitis* CBS 5773 (Table 1). *K. marxianus*, regarded as one of the fastest-growing yeasts, demonstrated surprisingly low μ among all phases in the used hydrolysate. Overall, *K. marxianus* CBS 6556 performed poorly on higher hydrolysate concentrations, reaching biomass of only 5 gL^–1^ with the highest μ of 0.03 h^–1^ in the glucose phase.

The diauxic growth found in most of the microorganisms during the microplate cultivation experiments can be explained by the carbon catabolite repression (CCR), wherein the presence of higher glucose concentrations in the environment, inhibits the consumption of other carbon sources. This phenomenon was observed by the sugar consumption profiles in the bioreactor experiments for all four strains studied. For *S. cerevisiae*, glucose represses the uptake and catabolism of other carbon sources when present in the extracellular medium via the inhibition of AMPK/Snf1, activation of PKA, and the regulation of transporter expression yeast casein kinases Yck1 and Yck2 (Simpson-Lavy and Kupiec, 2019). The glucose catabolite repression is considered one of the main challenges for efficient utilization and co-consumption of the diverse substrates in lignocellulosic hydrolysates (Hua et al., 2019; Simpson-Lavy and Kupiec, 2019). Metabolic engineering and adaptive laboratory evolution (ALE) have been used to alleviate the glucose catabolite repression in *S. stipitis* and *K. marxianus* (Slininger et al., 2015; Sharma et al., 2017; Hua et al., 2019; Kim et al., 2019). Different than expected based on the initial screening experiments, *L. starkeyi* DSM 70295 did not demonstrate a co-consumption of glucose and xylose but showed a preference to consume glucose and acetic acid first. Once glucose concentration was lower than 4.53 gL^–1^ and the acetic acid was completely depleted, this strain started to consume the other sugars. Glucose-induced carbon catabolite repression seemed to be abolished when low glucose levels were still present in the environment. Zhao et al. (2008) also reported sequential consumption of glucose and xylose by *L. starkeyi*, as only after the glucose concentration dropped from 48.9 to 3.5 gL^–1^, the xylose uptake was observed. Contrary to the findings of this work and the ones observed by Zhao et al. (2008) and Anschau et al. (2014) observed a co-consumption of the sugars when *L. starkeyi* was cultivated in hemicellulosic hydrolysate from sugarcane bagasse. In the initial screening experiments, the non-diauxic growth behavior might be explained by the low glucose concentration and by *L. starkeyi’s* similar growth rate on both, glucose and xylose.

*K. marxianus* is a fast-growing non-conventional yeast, able to consume a range of sugars as galactose, mannose, and lactose, but also pentose sugars, like xylose and arabinose (Nonklang et al., 2008; Goshima et al., 2013). This strain can also grow in hydrolysates of Japanese cedar and Eucalypt (Goshima et al., 2013). Although *K. marxianus* CBS 6556 showed the shortest lag phase and the highest μ during the glucose phase in the screening experiments, this strain did not demonstrate the same behavior in bioreactor experiments with a higher concentration of total sugars. It could be explained by the higher concentration of inhibitors during bioreactor experiments. Goshima et al. (2013), showed that *K. marxianus* grew poorly and produced very small amounts of ethanol from eucalyptus-based hydrolysates when compared with the growth and ethanol production when cultivated in Japanese cedar supplemented with yeast extract. The authors concluded that eucalyptus-based hydrolysates contain far more fermentation inhibitors than Japanese cedar and that *K. marxianus* has a low tolerance to inhibitors. Moreover, CCR and the presence of inhibitors, *K. marxianus* ability to uptake and convert xylose in hydrolysates containing xylose and glucose seems to be N-source dependent. Hua et al. (2019) demonstrated a varying performance by *K. marxianus* in hemicellulosic hydrolysates in terms of xylose consumption according to the type of nitrogen source used.

*S. stipitis* CBS 5773 had the second shortest lag phase in the plate reader experiments and a high μ during the glucose consumption. It is an important yeast because of its high native capacity to ferment xylose when compared with other yeasts. Therefore, *S. stipitis* genes like xylose reductase and xylitol dehydrogenase have been used to engineer xylose metabolism in other microorganisms (Jeffries, 2006; Jeffries et al., 2007). Nandal et al. (2020) analyzed the growth and fermentation of sugars obtained from rice straw hydrolysate by different yeasts and *S. stipitis* showed the highest sugar utilization and ethanol production. When this strain grew in rice straw hydrolysates containing 4.6% of total sugars, *S. stipitis* demonstrated glucose and xylose co-consumption with a glucose depletion after 96 h of fermentation while the xylose was depleted after 120 h of fermentation (Nandal et al., 2020). Despite *S. stipitis* CBS 5773 demonstrated to grow in plate reader experiments with a high μ and high maximum OD, in bioreactor experiments this strain demonstrated a long lag phase of almost 35 h. Further experiments demonstrated that the explanation for the longer lag phase when *S. stipitis* CBS 5773 grew in the bioreactor was probably due to glucose repression. Slininger et al. (2015) also reported long fermentation times (around 200 h) and CCR when cultivating *S. stipitis* in different hydrolysates. After performing an adaptation of the yeast, the authors reported reduced diauxic lag phase, improved rates, and shorter fermentation lengths (165 h).

In screening experiments, *R. toruloides* CCT 7815 had the highest value for maximum μ during the xylose consumption phase in the hydrolysate C5-Birch. This strain was previously adapted by Bonturi et al. (2017) in hemicellulosic sugarcane bagasse hydrolysate and showed upregulation of genes related to stress tolerance toward inhibitors and lipid accumulation. In bioreactor experiments, μ of *R. toruloides* CCT 7815 was the highest during the first growth phase. After 15 h of fermentation, when growth phase 2 started, the μ decreased possibly because of the nitrogen limitation. At this time, the C/N ratio was 26, the same C/N ratio was reported by Pinheiro et al. (2020) for the onset of nitrogen limitation. Also, at this time, all the sugars except xylose were depleted and the strain started to consume the latter. *R. toruloides* CCT 7815 depleted all sugars within 30 h of fermentation with a specific xylose consumption rate of 0.22 ± 0.02 g/gdw^∗^h. Costa et al. (2017) cultivated engineered *S. cerevisiae* with XR/XDH heterologous pathway in non-detoxified hemicellulosic hydrolysates (*Eucalyptus* and *Paulownia*) and agricultural residues (corn cob and wheat straw) and the most of them did not consume all the xylose presented within 72 or 96 h of fermentation.

*K. marxianus* and *S. stipitis* are industrially relevant ethanol producers under oxygen limiting conditions. As expected due to the fully aerobic environment, no ethanol was produced by *K. marxianus* CBS 6556 and *S. stipitis* CBS 5773. Only trace amounts of 2-PE and xylitol were produced by *K. marxianus* CBS 6556 and *S. stipitis* CBS 5773, respectively. Regarding lipid production in hydrolysates, although *L. starkeyi* DSM 70295 produced only 2.5 gL^–1^ (10% of lipid content), Brandenburg et al. (2016), achieved greater values of lipids production by this yeast (strain CBS 1807) using pH-regulated fed-batch cultivations. The authors used synthetic media during the batch phase, with glucose and xylose as carbon sources, and fed Birch hydrolysate, achieving production of 8.02 gL^–1^ (51.3%). These values are comparable with the production of the lipids achieved in this work by *R. toruloides* CCT 7815 (10.95 gL^–1^; 41%). Liu et al. (2020) achieved 1.01 gL^–1^ of lipids in the concentrated and decolorized wheat straw hemicellulosic hydrolysate using *R. toruloides*. Bonturi et al. (2017) and Lopes et al. (2020a) reported similar lipid titers of 3.3 and 3.7 gL^–1^ in eucalyptus and hemicellulosic sugarcane bagasse hydrolysates. Saini et al. (2020) reported higher lipid titers of 6.2 and 6.9 gL^–1^ in hydrolysates of hardwood (Maple) and softwood (Fir), respectively, in 96–104 h of cultivation. These values are lower than obtained in this work of a lipids titer of 10.95 ± 1.37 gL^–1^ in a C/N ratio of 45 (mol/mol) and less than 41 h of cultivation. Lopes et al. (2020b) achieved the highest lipid content in biomass (60%) using xylose and *R. toruloides* when using a C/N ratio of 120, almost three times higher than used in this study (C/N molar ratio = 45). A higher C/N ratio can therefore possibly increase the lipid accumulation, but also extend the cultivation time. Nonetheless, *R. toruloides* CCT 7815, *S. stipitis* CBS 5773, and *L. starkeyi* DSM 70295 can be suggested to be used as animal feed due to achieving over 20 gL^−1^ of biomass.

## Conclusion

Based on the cultivation conditions used in this work, *R. toruloides* CCT 7815 showed to be a potential candidate to be used in future biorefineries in terms of xylose consumption and conversion into different products when compared with other tested strains in this work. This strain was able to grow in high sugar and inhibitor concentrations present in the hydrolysate, it demonstrated a short lag phase, and consumed all the provided substrates in 30 h of fermentation. The other tested strains, *K. marxianus* CBS 6556, *S. stipitis* CBS 5773, and *L. starkeyi* DSM 70295, even in lower total sugars concentrations and consequently lower inhibitors concentrations, showed longer lag phases and some were not able to consume all the sugars present in the hydrolysate. It is important to investigate the potential of other strains and using metabolic engineering and ALE can further improve the consumption of hemicellulosic hydrolysates and direct the carbon toward the desired products.

## Data Availability Statement

The original contributions presented in the study are included in the article/Supplementary Material, further inquiries can be directed to the corresponding author/s.

## Author Contributions

PM, DA, NB, and P-JL designed the experiments. PM and DA performed the experiments. PM, DA, NB, and P-JL analyzed the data. All authors wrote and revised the manuscript.

## Conflict of Interest

The authors declare that the research was conducted in the absence of any commercial or financial relationships that could be construed as a potential conflict of interest.
