# Supplementary material for: Screening and Growth Characterization of Non-conventional Yeasts in a Hemicellulosic Hydrolysate
Source: Front Bioeng Biotechnol. 2021 Apr 29;9:659472. doi: 10.3389/fbioe.2021.659472 (PMC8116571; doi:10.3389/fbioe.2021.659472)
Supplement: Supplementary file 3 [file Image_1.PDF]

Supplementary Material

Supplementary Figure

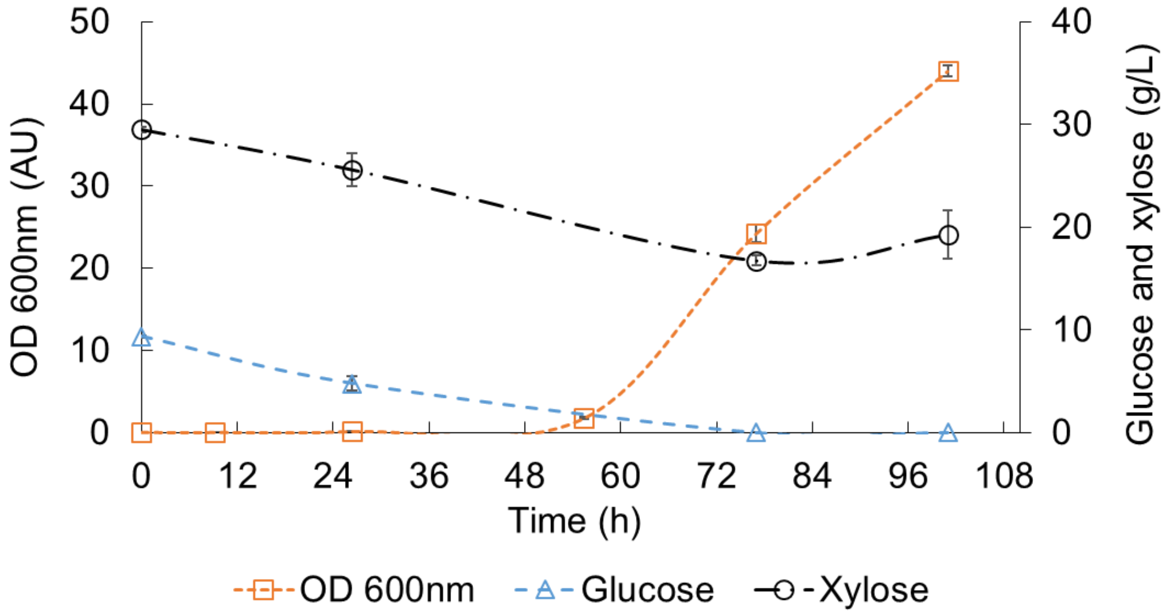

Supplementary Figure 1. Growth of *S. stipitis* CBS 5773 in minimal medium containing 50 gL-1 of glucose plus xylose (1:3.5 ratio) and a C/N molar ratio of 10 (same used in bioreactor experiments). The cultivation was carried out in triplicates using 125 mL shake flasks containing a working volume of 25 mL and the yeast was incubated at 30 ºC and 200 rpm. Samples were taken regularly for OD 600 nm measurement and sugars analysis. Errors are expressed in standard deviation.
